# Imaging of the intrinsic muscles of the hand – part I: high-resolution ultrasound and 3T MRI appearance of symptomatic anatomical variants

**DOI:** 10.1055/a-2761-4259

**Published:** 2026-01-08

**Authors:** Hicham Bouredoucen, Sana Boudabbous, Pierre-Alexandre Poletti, Lokmane Taihi

**Affiliations:** 1 27230 Imaging and Medical Informatics, Geneva University Hospitals, Geneva, Switzerland; 2 70492 Medical Imaging, University Hospital Saint-Luc, Brussels, Belgium

**Keywords:** Intrinsic muscles of the hand, Anatomical variants, Carpal tunnel syndrome, Guyon’s Canal Syndrome, MRI imaging

## Abstract

**Background:**

The intrinsic muscles of the hand (IMH) include the thenar muscles, hypothenar muscles, lumbrical muscles, dorsal interosseous muscles (DIOM), and ventral interosseous muscles (VIOM). The thenar muscles consist of the abductor pollicis brevis (APB), opponens pollicis (OPP), flexor pollicis brevis (FPB), and adductor pollicis (ADP). The hypothenar muscles comprise the abductor digiti minimi (ADM), flexor digiti minimi (FDM), and opponens digiti minimi (ODM). Numerous anatomical variants of the IMH exist – including the accessory abductor digiti minimi (aADM), adductor hypothenar muscle, extensor digitorum brevis manus (EDBM), lumbrical muscle (LM) variants, and accessory flexor digitorum superficialis of the index finger. Although these variants are common, they can cause symptoms, especially in nerve compression syndromes such as carpal tunnel syndrome (CTS) from median nerve (MN) compression or Guyon’s canal syndrome from ulnar nerve (UN) compression. Knowledge of these variants and their imaging characteristics facilitates understanding of related pathologies and contributes to improved therapeutic management. These muscle variants are diagnosed using high-resolution ultrasound (US) and magnetic resonance imaging (MRI).

**Method:**

This review provides a comprehensive overview of the normal anatomy of the IMH, their anatomical variants, and their imaging features. High-resolution US is the primary modality for studying the IMH, while high-field 3T MRI offers excellent spatial resolution and contrast.

**Results and Conclusion:**

Understanding the anatomy and anatomical variants of the IMH is essential for accurately assessing both normal and pathological conditions using US and MRI.

**Key Points:**

- Variants of the intrinsic hand muscles can be reliably diagnosed by ultrasound and high-resolution MRI.
- Accessory abductor digiti minimi (aADM) may cause compression of the ulnar nerve within the Guyon’s canal.
- Extensor digitorum brevis manus (EDBM) can mimic a dorsal pseudotumoral soft-tissue mass.
- Variants of the lumbrical muscles may compress the median nerve within the carpal tunnel.
- Accessory flexor digitorum superficialis indicis can simulate a tumor or cause carpal tunnel syndrome.

**Citation Format:**

- Bouredoucen H, Boudabbous S, Poletti P et al. Imaging of the intrinsic muscles of the hand - part I: high-resolution ultrasound and 3T MRI appearance of symptomatic anatomical variants. Rofo 2025; DOI 10.1055/a-2761-4259

## Zusammenfassung

**Hintergrund:**

Die intrinsischen Handmuskeln (IHM) umfassen die Thenarmuskeln, Hypothenarmuskeln, Lumbrikalmuskeln, dorsalen Interossären Muskeln (DIOM) und ventralen Interossären Muskeln (VIOM). Die Thenarmuskeln bestehen aus dem Musculus abductor pollicis brevis (APB), Musculus opponens pollicis (OPP), Musculus flexor pollicis brevis (FPB) und Musculus adductor pollicis (ADP). Die Hypothenarmuskeln umfassen den Musculus abductor digiti minimi (ADM), Musculus flexor digiti minimi (FDM) und Musculus opponens digiti minimi (ODM). Anatomische Varianten der IHM, wie der akzessorische Musculus abductor digiti minimi (aADM), der adductor hypothenaris, der Musculus extensor digitorum brevis manus (EDBM), Varianten der Lumbrikalmuskeln (LM) sowie der akzessorische Musculus flexor digitorum superficialis indicis, sind zahlreich, aber nicht selten. Obwohl diese Varianten häufig vorkommen, können sie Symptome verursachen, insbesondere bei Nervenkompressionssyndromen wie dem Karpaltunnelsyndrom (KTS) durch Kompression des Nervus medianus (NM) oder dem Guyon-Kanal-Syndrom durch Kompression des Nervus ulnaris. Das Wissen über diese Varianten und deren bildgebende Diagnostik erleichtert das Verständnis der Pathologien und trägt zu einer besseren therapeutischen Behandlung bei. Die Diagnose erfolgt mittels hochauflösender Ultraschalluntersuchung (US) und Magnetresonanztomografie (MRT).

**Methode:**

Dieser Übersichtsartikel bietet eine umfassende Darstellung der normalen Anatomie der IHM, ihrer anatomischen Varianten und deren bildgebender Merkmale. Hauptdiagnoseverfahren sind hochauflösender Ultraschall und 3-Tesla MRT, die exzellente räumliche Auflösung und Kontrast bieten.

**Ergebnisse und Schlussfolgerung:**

Die Kenntnis der Anatomie und der anatomischen Varianten der IHM ist essenziell für die Beurteilung normaler und pathologischer Befunde mittels Ultraschall und MRT.

**Kernaussagen:**

- Intrinsische Handmuskelvarianten können zuverlässig mittels Ultraschall und hochauflösender MRT diagnostiziert werden.
- Der akzessorische Musculus abductor digiti minimi (aADM) kann den Ulnarisnerv im Guyon-Kanal komprimieren.
- Der Musculus extensor digitorum brevis manus (EDBM) simuliert eine pseudotumorale Weichteilraumforderung am Handrücken.
- Varianten der Lumbrikalmuskeln können den Nervus medianus im Karpaltunnel komprimieren.
- Der akzessorische Musculus flexor digitorum superficialis indicis kann einen Tumor simulieren oder ein Karpaltunnelsyndrom verursachen.

## Abbreviations

aADM: Accessory abductor digiti minimi
ADM: Abductor digiti minimi
ADP: Adductor pollicis
APB: Abductor pollicis brevis
CT: Carpal tunnel
CTS: Carpal tunnel syndrome
DIOM: Dorsal interosseous muscles
EDBM: Extensor digitorum brevis manus
EI: Extensor indicis
FDM: Flexor digiti minimi
FPB: Flexor pollicis brevis
FPL: Flexor pollicis longus
GC: Guyon’s canal
IMH: Intrinsic muscles of the hand
IOM: Interosseous muscles
L1: First lumbrical muscle
L2: Second lumbrical muscle
L3: Third lumbrical muscle
L4: Fourth lumbrical muscle
LM: Lumbrical muscle
MC: Metacarpal
MC1: First metacarpal
MN: Median nerve
MRI: Magnetic resonance imaging
ODM: Opponens digiti minimi
OPP: Opponens pollicis
P1: First phalanx
PBM: Palmaris brevis
UA: Ulnar artery
UN: Ulnar nerve
US: Ultrasound
VIOM: Ventral interosseous muscles

## 1. Introduction

Anatomical muscle variations are relatively common in the hand and wrist. While often asymptomatic, some variants may compress adjacent neurovascular structures – particularly the median or ulnar nerve – leading to tunnel syndromes such as CTS or Guyon’s canal syndrome. These variations can be reliably identified using high-resolution US and MRI.

### Methodology

For this review, a systematic literature search was conducted to ensure a comprehensive and reproducible overview of anatomical variants of the intrinsic hand muscles. The databases that were consulted were PubMed/MEDLINE, Scopus, and Web of Science, with the search including all relevant articles published up to the end of 2024.

The search strategy combined keywords such as “intrinsic hand muscles”, “thenar muscles”, “hypothenar muscles”, “lumbrical muscles”, “variants of the flexor digitorum superficialis”, “accessory hand muscles”, “carpal tunnel syndrome”, “Guyon’s canal syndrome”, “anatomical variations”, “muscle anomalies”, and “hand ultrasonography”. Only articles published in English were considered.

Inclusion criteria comprised original research articles, systematic reviews, meta-analyses, anatomical, imaging, and surgical studies, as well as clinical case reports focusing on anatomical variants and their clinical implications for the intrinsic hand muscles. Articles unrelated to the topic, lacking significant anatomical or clinical data, or not available in full text were excluded.

This approach allowed for rigorous and methodical selection of relevant literature to support the scope and conclusions of this review.

## 2. Normal anatomy of the intrinsic muscles of the hand

**Fig. 1:**
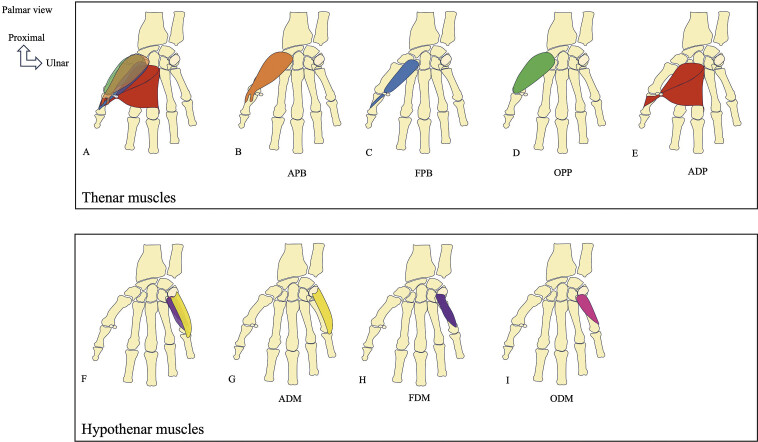
Schematic drawings showing the topography and bony insertions of the thenar and hypothenar muscles of the hand. Thenar muscles: Abducter pollicis brevis (APB), flexor pollicis brevis (FPB), opponens pollicis (OPP), adductor pollicis (ADP). Hypothenar muscles: Opponens digiti minimi (ODM), flexor digiti minimi (FDM), abductor digiti minimi (ADM).

Hand movements are the result of interactions between intrinsic and extrinsic muscles. The intrinsic muscles originate in the hand itself ( Fig. 1 ), and the insertion of the extrinsic hand muscles is primarily located in the forearm. The intrinsic muscles of the hand (IMH) include the thenar muscles, hypothenar muscles, lumbrical muscles, and dorsal and palmar interosseous muscles. The hand is generally described as being composed of 10 myotendinous compartments bounded by fascia, four dorsal interosseous, three ventral interosseous, the thenar, the hypothenar, and the ADP. Anatomical studies suggest interindividual variability of these compartments.

### 2.1. Thenar muscles

The thenar muscles include the APB, OPP, FPB, and ADP ( Fig. 1 ). The interaction between the intrinsic and extrinsic thenar musculature controls thumb movements, enabling precise pinching and a powerful grip. The primary function of the thumb is to oppose the index finger and other fingers. The thumb functions with a balance between movement on the one hand and stability and joint congruity on the other. The musculotendinous system ensures active stability during movement.

**Fig. 2:**
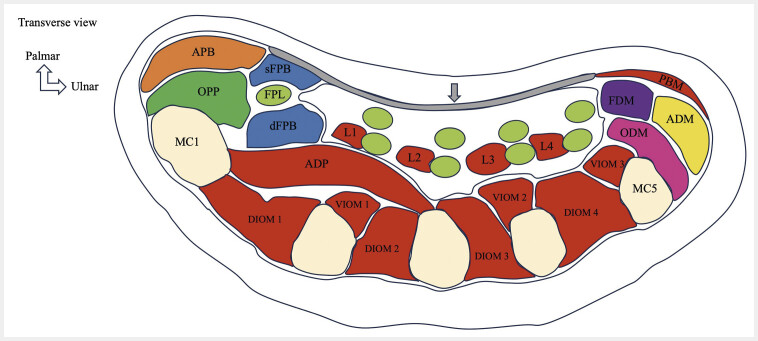
Schematic drawings showing the intrinsic muscles of the hand on a transverse view at the level of the metacarpals. Thenar muscles: Abductor pollicis brevis (APB), opponens pollicis (OPP), flexor pollicis brevis (FPB), superficial (sFPB), deep (dFPB), adductor pollicis (ADP). Hypothenar muscles: Palmaris brevis muscle (PBM), flexor digiti minimi (FDM), opponens digiti minimi (ODM), abductor digiti minimi (ADM). Lumbrical muscles: First lumbrical muscle (L1), second lumbrical muscle (L2), third lumbrical muscle (L3), fourth lumbrical muscle (L4). Interosseous muscles: First ventral interosseous muscle (VIOM 1), second ventral interosseous muslce (VIOM 2), third ventral interosseous muscle (VIOM 3), first dorsal interosseous muscle (DIOM 1), second dorsal interosseous muslce (DIOM 2), third dorsal interosseous muscle (DIOM 3), fourth dorsal interosseous muscle (DIOM 4). Flexor pollicis longus (FPL), palmar aponeurosis (gray arrow). First metacarpal (MC1), fifth metacarpal (MC5).

**Fig. 3:**
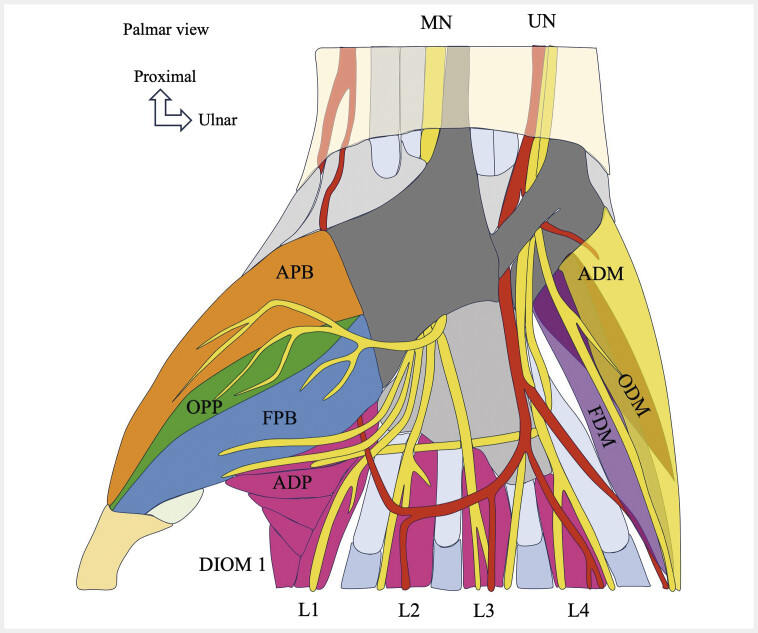
Schematic drawings showing the intrinsic muscles of the hand and their innervations in a palmar view. Thenar muscles: Abductor pollicis brevis (APB), opponens pollicis (OPP), flexor pollicis brevis (FPB), adductor pollicis (ADP). Hypothenar muscles: Flexor digiti minimi (FDM), opponens digiti minimi (ODM), abductor digiti minimi (ADM). LMs: First lumbrical muscle (L1), second lumbrical muscle (L2), third lumbrical muscle (L3), fourth lumbrical muscle (L4). First dorsal interosseous muscle (DIOM 1). Median nerve (MN). Ulnar nerve (UN).

**Fig. 4:**
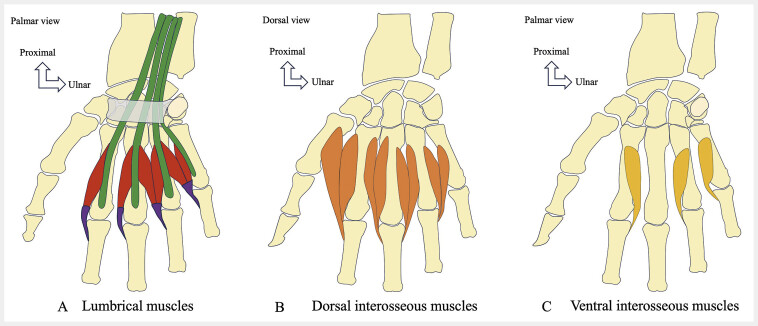
Schematic drawings showing the lumbrical and interosseous muscles of the hand. There are four LMs ( **A** ), arising from the tendons of the deep digital flexor muscle, distal to the carpal tunnel. The LM tendons fuse with the IOM and terminate on the radial lateral band of the extensor tendon. There are four DIOM ( **B** ), which insert proximally via two groups of fibers on the lateral surface of the MC. There are three VIOM ( **C** ), which insert proximally on the palmar half of the lateral surface of the MC furthest from the middle finger. The IOM have distal capsular insertions on the distal portion of the palmar plate of the metacarpophalangeal joint, sometimes bony on the tubercle of the P1 base, and on the extensor apparatus.

***The APB*** is a flattened, triangular muscle and is the most superficial and radial of the thenar eminence muscles, located subcutaneously on the radial and proximal surface of the thenar eminence ( Fig. 2 ). It extends from the first row of the carpus to the first phalanx (P1) of the thumb ( Fig. 1 B). It arises from the superolateral portion of the anterior surface of the transverse carpal ligament ( Fig. 3 ), the scaphoid tubercle, and the abductor pollicis longus tendon. Occasionally, some fibers originate from the trapezium 1 . The muscle is divided into two lamellae. The APB is innervated by the recurrent motor branch of the MN (95%) ( Fig. 3 , Fig. 4 ), rarely by the UN (2.5%) or by a dual innervation (2%) 1 2 .

***The FPB*** is a triangular muscle located medial to the palmar surface of the first metacarpal (MC1) and anterior to the lateral portion of the ADP muscle ( Fig. 2 ). It extends from the second row of carpal bones to the P1 of the thumb ( Fig. 1 C). It is composed of two bundles whose union forms the fleshy body, which has a concave groove on its upper part where the tendon of the FPL runs ( Fig. 2 ). The recurrent motor branch of the MN crosses the FPB ( Fig. 2 ). The innervation of the FPB can be highly variable. The superficial head is generally innervated by the recurrent motor branch of the MN, while the deep head is generally innervated by the deep motor branch of the UN ( Fig. 3 , Fig. 4 ). Both heads may be innervated by the MN alone or by the UN exclusively, or both heads may be doubly innervated. The Cannieu-Riche anastomosis, or the thenar loop, is a connection between the deep motor branch of the UN and the recurrent motor branch of the MN found in up to 77% of dissections 3 .

***The OPP*** is superficial, triangular, and lies beneath the APB and lateral to the superficial fasciculus of the FPB ( Fig. 2 ). It extends from the second row of carpal bones to the MC1 ( Fig. 1 D). It arises from the carpometacarpal joint capsule, the trapezium tubercle, and the anterior surface of the transverse carpal ligament ( Fig. 3 ). It runs inferiorly and laterally, overlying the MC1, and extends to insert along the palmar radial length of MC1 1 .

The OPP is often innervated by the recurrent branch of the MN (83%) ( Fig. 3 , Fig. 4 ), sometimes by the UN (9%), or by a dual median and ulnar innervation (7.5%) 1 .

***The ADP*** is the deepest thenar muscle. It is triangular, flattened, and located anterior to the first two interosseous spaces ( Fig. 2 ). It extends from the carpal mass of the second and third MC to P1 of the thumb ( Fig. 1 E). The ADP is composed of two heads, oblique and transverse, which converge to insert at the base of P1 of the thumb. The first dorsal interosseous muscle lies posterior to the ADP, and together, these two muscles constitute the majority of the first interosseous space 1 ( Fig. 2 ). On the palmar side, the muscle is crossed by the flexor tendons of the index finger and the first lumbrical muscle (L1) ( Fig. 2 ). The ADP is mainly innervated by the deep motor branch of the UN ( Fig. 3 , Fig. 4 ). In 2% of cases, all thenar muscles, including the ADP, are innervated solely by the MN 1 .

### 2.2. Hypothenar muscles

The hypothenar muscles include the ADM, FDM, and ODM. They coordinate the movement of the little finger.

***The palmaris brevis (PBM)*** is a flattened muscle lamella of variable morphology located in the subcutaneous tissue on the ulnar aspect of the palm, on the surface of the hypothenar eminence ( Fig. 2 ). It originates from the palmar aponeurosis ( Fig. 2 ) and inserts into the hypothenar fascia and the dermis along the ulnar margin of the hypothenar eminence 4 . It is separated from the other muscles of the hypothenar eminence by the palmar fascia. The UN and vessels are positioned beneath the PBM 4 . The muscle is innervated by the superficial branch of the UN.

***The ADM*** has three origins: bony from the pisiform muscle ( Fig. 1 G), tendinous from the flexor carpi ulnaris tendon, and ligamentous from the pisohamatum ligament 5 . The ADM often has different insertions: in the ulnar aspect of the base of P1, in the extensor apparatus 6 , and in the joint capsule of the little finger metacarpophalangeal. The muscle is innervated by the deep branch of the UN.

***The FDM*** arises from the hamulus of the hamate, the ulnar portion of the flexor retinaculum, and the radial portion of the pisiform muscle ( Fig. 1 H). In most cases, there is one muscle belly. The proximal insertion of the ADM and FDM delimits the “pisohamate hiatus”, corresponding to a narrow passage through which the motor branch of the UN reaches the deep part of the palm. It is innervated by the deep branch of the UN.

***The ODM*** also arises from the hamulus of the hamate and the flexor retinaculum ( Fig. 1 I). It has two layers with distinct muscular origins. It inserts on the distal ulnar surface and the proximal ulnar surface of the shaft of the little finger metacarpal. The ODM lies deep with respect to the other two hypothenar muscles. It differs from the ADM and FDM in that it is the only one to insert into the little finger MC ( Fig. 1 I). The motor branch of the UN innervates the ODM muscle.

### 2.3. Lumbrical and interosseous muscles

***The LMs*** ( Fig. 4 A) arise from the tendons of the deep digital flexor muscle, distal to the carpal tunnel. There are four of them. The proximal insertions of the first LM are on the radial edge of the deep digital flexor tendon of the index finger, and those of the second LM are on the radial edge of the deep digital flexor tendon of the middle finger. The third and fourth LMs have a bipennate origin: the deep digital flexor tendons of the middle and ring fingers for the third LM, and the deep digital flexor tendons of the ring and little fingers for the fourth LM. The tendon passes through the palmar surface of the deep transverse metacarpal ligament, then runs to the dorsal surface of the finger, fusing with the interosseous muscles (IOM), and ending on the radial lateral band of the extensor tendon. They are extensors of the proximal interphalangeal and distal interphalangeal joints. The first and second LMs are innervated by the MN, while the third and fourth LMs are generally innervated by the deep branch of the UN ( Fig. 3 ). There are some variations in innervation.

There are four ***DIOM*** ( Fig. 4 B **, C** ). They have proximal insertions via two groups of fibers, a first group on the lateral surface of the MC closest to the middle finger, and a second group on the dorsal half of the lateral surface of the MC furthest from the middle finger. The middle finger represents the axis of the hand. There are three ***VIOM*** . They have proximal insertions on the palmar half of the lateral surface of the MC furthest from the middle finger. The IOM have distal insertions, deep insertions, capsular on the distal part of the metacarpophalangeal palmar plate, and sometimes bony on the tubercle of the P1 base, and a superficial insertion on the extensor apparatus. The IOM are innervated by the deep branch of the UN.

## 3. Ultrasound of the intrinsic muscles of the hand

High-resolution US is the first-line imaging modality for studying IMH pathologies. High- and ultrahigh-frequency US, using transducers with frequency bands above 20 MHz, allows for the investigation of IMHs with excellent resolution due to their superficial position. It allows for analysis by performing dynamic maneuvers, examining the anatomical structure during stress tests, and simulating conditions that reproduce clinical symptoms 7 . Furthermore, a comparative study of contralateral IMH is easily performed using US.

### Thenar muscles ( Fig. 5 A, B)

**Fig. 5:**
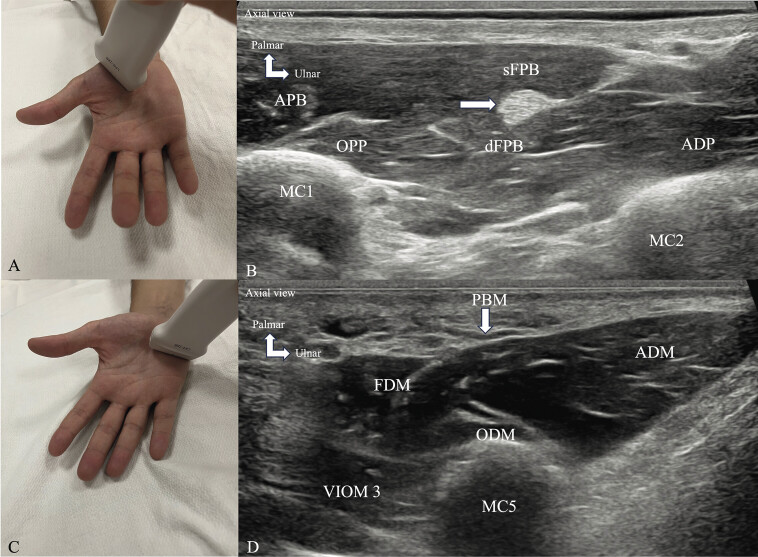
Normal ultrasound appearance of thenar muscles ( **A, B** ) and hypothenar muscles ( **C, D** ). The probe is placed perpendicular to the long axis of the MC1 ( **A** ), the four muscles of the thenar eminence are identified from superficial to deep ( **B** ): the APB on the radial side, the superficial belly of the FPB (sFPB) on the ulnar side, the OPP on the radial side, the deep belly of the FPB (dFPB), the tendon of the flexor pollicis longus (white arrow) is interposed between the two bellies of the FPB, and the ADP which constitutes the deepest thenar muscle. The probe is placed perpendicular to the long axis of of the fifth MC (MC5) ( **C** ), the muscles of the hypothenar eminence are identified from the surface to the depth ( **D** ): The palmaris brevis (PBM) is a thin superficial muscle, located in the subcutaneous tissue of the hypothenar eminence, the ADM on the ulnar side of the metacarpal shaft, the FDM on the radial side, the ODM is located on the radial side, deep to the two previous muscles. First metacarpal (MC1), second metacarpal (MC2).

The probe is placed perpendicular to the long axis of the MC1, and the four muscles of the thenar eminence are identified from superficial to deep: the APB on the radial side of the MC1 shaft, the superficial belly of the FPB on the ulnar side, the OPP on the radial side, the deep belly of the FPB, the FPL tendon interposed between the two bellies of the FPB, and the ADP, which constitutes the deepest thenar muscle.

### Hypothenar muscles ( Fig. 5 C, D)

The probe is placed perpendicular to the long axis of the little finger MC. The muscles of the hypothenar eminence are identified from superficial to deep: the PBM is a thin superficial muscle located in the subcutaneous tissue of the hypothenar eminence; the ADM is located on the ulnar side of the MC shaft; the FDM is located on the radial side; and the ODM is located on the radial side, deep with respect to the two preceding muscles.

### Lumbrical muscles ( Fig. 6 C, D)

**Fig. 6:**
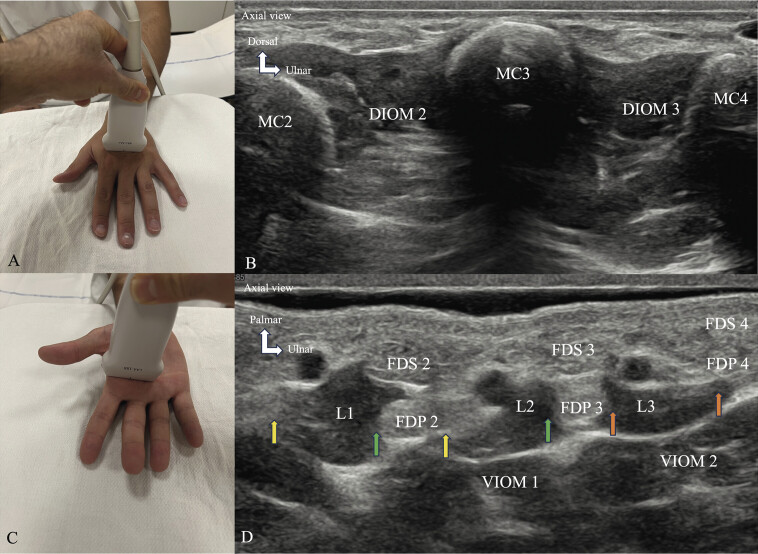
Normal ultrasound appearance of interosseous and lumbrical muscles. The probe is placed transversely at the level of the metacarpal shafts on the dorsal aspect of the hand to identify the DIOM ( **A** ), which are located in the four interdigital spaces ( **B** ). To visualize the VIOM, the probe is placed transversely in the mid-palm ( **C, D** ). The VIOM are located under the flexor tendons and the LMs in the second, third, and fourth interosseous spaces. The ventral portion of the first interosseous space is occupied by the ADP. Due to their small size, LMs are best assessed using US probes with frequency bands around 20 MHz. The probe should be placed in the middle of the palm, oriented along the short axis of the metacarpal shafts. The LMs ( **C, D** ) are identified in the intermetacarpal spaces between the flexor tendons. While L1 is easily assessed along its entire length up to the distal insertion, L2, L3, and L4 are only partially accessible on US distal to the deep intermetacarpal ligament due to their deep location between the heads of the MC. Short-axis US images obtained at the distal third of the MCs show the position of L1, L2, and L3 between the tendinous glides of the index, middle, and ring fingers of the flexor digitorum superficialis (FDS 2, FDS 3, and FDS 4), and the flexor digitorum profundus (FDP 2, FDP 3, and FDP 4). Note the unipinnate origin of L1 and L2 from the radial side of the tendon glide for the middle of the FDP. The ulnar edges of L1 and L2 are inserted onto the radial edges of FDP 2 and FDP 3, respectively (green arrows), but their radial edges are free and surrounded by fat (yellow arrows). Note the bipinnate origin of L3 inserted onto the ulnar side of FDP 3 and onto the radial side of FDP 4 (orange arrows). Second metacarpal (MC2), third metacarpal (MC3), fourth metacarpal (MC4).

The probe is placed in the mid-palm perpendicular to the long axis of the MC shafts. The LMs are located between the flexor tendons in the intermetacarpal spaces. The first LM is identified along its entire length; it is located on the radial side of the second MC and is easily examined due to the width of the first intermetacarpal space. The second, third, and fourth LMs are partially explored. Their deep location in the last three intermetacarpal spaces limits probe positioning.

### VIOM and DIOM ( Fig. 6 A, B, C, D)

The probe is placed transversely at the level of the MC shafts on the dorsal aspect of the hand to identify the DIOM located in the four interdigital spaces. To visualize the palmar interosseous muscles, the probe is placed transversely in the medial part of the palm. The VIOM are located under the flexor tendons and the LMs in the second, third, and fourth interosseous spaces. The palmar portion of the first interosseous space is occupied by the ADP.

## 4. MRI of the intrinsic muscles of the hand

**Fig. 7:**
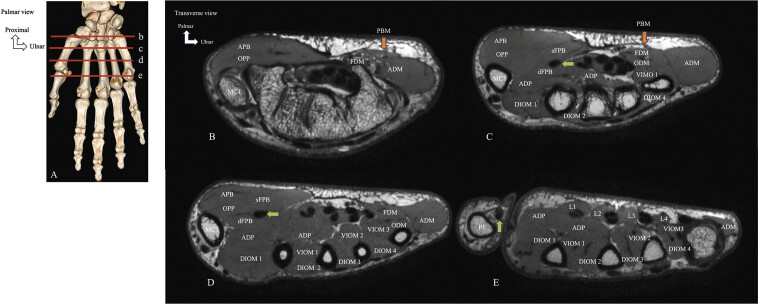
Normal appearance of the intrinsic muscles of the hand on transverse MRI. Levels of the different cross sections, CBCT of the hand, volumetric rendering reconstructions ( **A** ). T1-weighted TSE MRI, transverse sections ( **B, C, D, E** ). Transverse section at the trapeziometacarpal joint and the hamulus of the hamate ( **B** ). Transverse section at the proximal third of the metacarpals ( **C** ). Transverse section at the distal third of the MC1 and the shaft of the metacarpals of the long fingers ( **D** ). Transverse section at the interphalangeal joint of the thumb and the distal third of the metacarpals of the long fingers ( **E** ). Abductor pollicis brevis (APB), opponens pollicis (OPP), flexor pollicis brevis (FPB), superficial (sFPB), deep (dFPB), adductor pollicis (ADP), palmaris brevis muscle (PBM), flexor digiti minimi (FDM), opponens digiti minimi (ODM), abductor digiti minimi (ADM). First lumbrical muscle (L1), second lumbrical muscle (L2), third lumbrical muscle (L3), fourth lumbrical muscle (L4), first ventral interosseous muscle (VIOM 1), second ventral interosseous muslce (VIOM 2), third ventral interosseous muscle (VIOM 3), first dorsal interosseous muscle (DIOM 1), second dorsal interosseous muslce (DIOM 2), third dorsal interosseous muscle (DIOM 3), fourth dorsal interosseous muscle (DIOM 4), flexor pollicis longus tendon (green arrow), first metacarpal (MC1), first phalanx (P1).

MRI is an excellent modality for analyzing the IMH ( Fig. 7 ). MRI should be performed on high magnetic field MRI machines (at least a 1.5T magnet, but preferably a T3 magnet should be used), with dedicated multichannel coils. The patient should be positioned in the “Superman position” with the arm above the head. This position will allow alignment of the region of interest within the scanner isocenter. However, this position can be uncomfortable and difficult to maintain, potentially resulting in motion artifacts. If the patient is not too large and the previous positioning is not tolerated, the hand can be photographed with the patient in the supine position and the arm at the side of the body. Skeletal muscles are assessed on T1-weighted fast spin echo sequences ( Fig. 7 ). Normal muscles have an intermediate signal intensity compared to the high signal intensity of fat or the low signal intensity of cortical bone, and a higher signal intensity than that of water. In the hand, however, differentiating between the different IMH is sometimes difficult due to two factors, i.e., the small size of the interposed fat and partial volume artifacts caused by the obliquity of the IMH relative to the axial plane of the images which can cause disappearance of the muscle contours. On contrast-enhanced T1-weighted sequences, normal muscles show little or no enhancement 8 . T2-weighted sequences optimized for more sensitive detection of edema are used. To detect pathological disorders such as neurogenic edema or myositis, various fat signal suppression techniques can be used: frequency pre-saturation (CHESS), inversion recovery (STIR), and the Dixon technique. To obtain optimal images with consistent and more homogeneous suppression, the STIR and Dixon techniques are preferred over the frequency pre-saturation technique 9 . The basic MRI protocol for IMH analysis should include T1-weighted sequences (anatomical study, search for fat involution or muscle atrophy) and T2-weighted sequences optimized for fat saturation or STIR (search for denervation edema or myositis). Enhanced T1-weighted sequences do not help to diagnose denervation edema or myositis 10 , but they are useful for other diagnoses (infection, necrosis, tumors).

## 5. Anatomical variants of the intrinsic muscles of the hand

### 5.1. Variants of the APB, FPB, OPP, PBM, ADM, and FDM

Variants of the APB are represented by additional heads or variable attachments 1 . Variants of the FPB are represented by the absence of the deep head 11 , an accessory deep head, fusion of the superficial head of the FPB with the OPP 1 , or a FPB fascicle originating from the ulnar surface of the thumb MC that inserts on the ulnar base of P1. Variants of the OPP include accessory heads of the muscle, fusion with the FPB, and the OPP muscle is rarely absent 1 . Anatomical variations of the PBM muscle include a hypertrophied PBM 4 , a deep PBM, or a superficial ulnar artery (UA) to the muscle. A variant of the PBM may be involved in distal NU neuropathy through nerve compression, leading to weakened grip and muscle atrophy 12 . Variants of the ADM are represented by the aADM, variations in the size of this muscle, and an absent ADM. The FDM may present with variants including an absent FDM with concomitant hypertrophy of the ADM, an FDM not fusing with the ADM, and in these cases, the FDM has an independent insertion distally into the palmar aspect of the head of the fifth MC 5 6 .

### 5.2. Accessory abductor digiti minimi muscle (aADM)

**Fig. 8:**
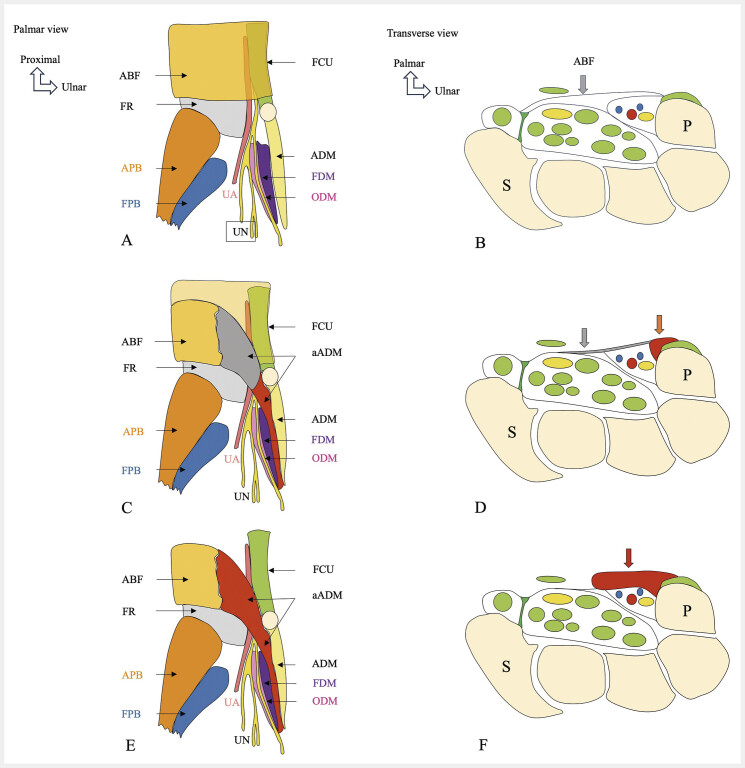
Normal anatomical presentation ( **A, B** ) and schematic representations of accessory abductor digiti minimi muscle (aADM) variants ( **C–F** ). Schematic illustrations depicting typical and variant anatomy of the aADM. Normal configuration ( **A, B** ): the aADM is absent. The roof of Guyonʼs canal is typically formed by the superficial palmar aponeurosis, fibers from the extensor retinaculum, the flexor carpi ulnaris, and the palmaris brevis muscle. In the distal forearm, the proximal antebrachial fascia passes beneath the palmaris longus tendon and continues as the palmar aponeurosis. Type I aADM (fascial-type) ( **C, D** ): the muscle belly is restricted to Guyon's canal (orange arrow, **D** ), with a fascial origin from the proximal antebrachial fascia of the wrist (gray arrow, **D** ), located just proximal to the flexor retinaculum. Type II aADM (muscular-type) ( **E, F** ): this variant features a muscle belly positioned deep with respect to the fascia, adjacent to the neurovascular bundle (red arrow, **F** ). Abbreviations: accessory abductor digiti minimi muscle (aADM), antebrachial fascia (ABF), flexor retinaculum (FR), flexor carpi ulnaris (FCU), abductor pollicis brevis (APB), flexor pollicis brevis (FPB), flexor digiti minimi (FDM), opponens digiti minimi (ODM), abductor digiti minimi (ADM), ulnar artery (UA), ulnar nerve (UN), scaphoid (S), pisiform (P).

**Fig. 9:**
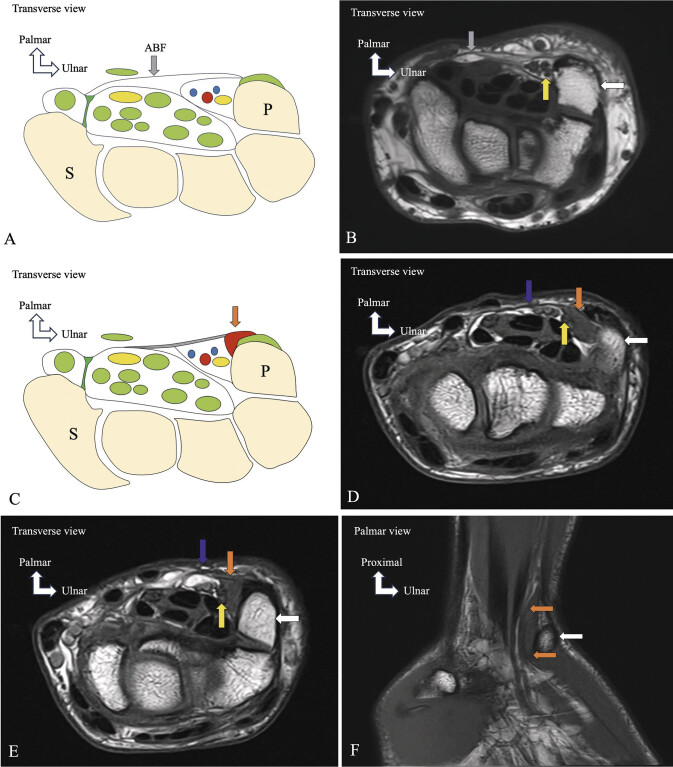
Normal anatomical presentation and type I variant of the accessory abductor digiti minimi muscle (fascial-type). Schematic drawings ( **A, C** ) and corresponding MRI images ( **B, D–F** ). Normal anatomy ( **A, B** ): no accessory abductor digiti minimi muscle (aADM) is present. The typical roof of Guyon's canal is composed of the superficial palmar aponeurosis, fibers from the extensor retinaculum, the flexor carpi ulnaris, and the palmaris brevis muscle. In the distal forearm, the proximal antebrachial fascia (ABF, gray arrows) courses beneath the palmaris longus and continues as the palmar aponeurosis.Type I aADM (fascial-type) ( **C–F** ): the muscle arises at the junction between the distal antebrachial fascia and the flexor retinaculum, near the palmar carpal ligament and the radial border of the pisiform, at the proximal edge of Guyonʼs canal. The fascial component is located proximal to the canal, while the muscular portion emerges within the canal and extends distally. MRI: Axial ( **D, E** ) and coronal ( **F** ) T1-weighted TSE images show the muscle belly (orange arrows), fascial component (blue arrows), ulnar nerve (yellow arrows), and pisiform (white arrows). Schematic drawing (C) illustrates the aADM (orange arrow) with anatomical landmarks including the pisiform (P) and scaphoid (S).

**Fig. 10:**
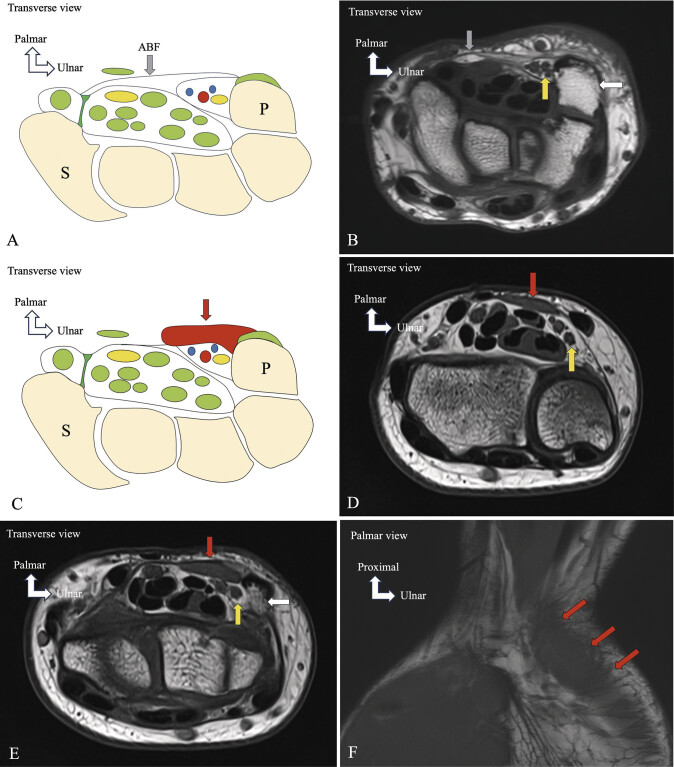
Normal anatomical presentation and type II variant of the accessory abductor digiti minimi muscle (aADM muscular-type). Schematic drawings ( **A, C** ) and corresponding MRI images ( **B, D–F** ). Normal anatomy ( **A, B** ): No accessory abductor digiti minimi muscle (aADM) is present. The typical roof of Guyon's canal is formed by the superficial palmar aponeurosis, fibers from the extensor retinaculum, the flexor carpi ulnaris, and the palmaris brevis muscle. In the distal forearm, the proximal antebrachial fascia (ABF, gray arrows) lies deep with respect to the palmaris longus tendon and continues into the palmar aponeurosis. Type II aADM ( **C–F** ): This variant arises from the antebrachial fascia in the distal forearm and is entirely muscular throughout its course. The muscle lies in close proximity to the local flexor tendons, the scaphoid (S), and the pisiform (P). Schematic drawing highlights the aADM muscular-type (red arrow, **C** ). Axial (proximal: **D** ; distal: **E** ) and coronal ( **F** ) T1-weighted TSE MR images demonstrate the aADM (red arrows, **D–F** ), ulnar nerve (yellow arrows, **D, E** ), and pisiform (white arrow, **E** ).

The aADM is the most common accessory hypothenar muscle, with a prevalence of up to 25% 13 . It is more common in men than in women (56% vs. 44%) 13 , and the anteroposterior muscle dimension is greater in males 13 . It can be bilateral in up to 50% of cases 14 . The aADM may originate from the antebrachial fascia, the flexor retinaculum, or the palmaris longus tendon 15 . It typically attaches to the pisiform bone, flexor carpi ulnaris, and pisohamate ligament, then runs over the ulnar neurovascular bundle within the Guyon’s canal (GC), before inserting either into the ADM or adjacent to it, on the ulnar base of the P1 of the fifth digit and the extensor hood 5 . Two anatomical types have been described ( Fig. 8 ): type I (fascial variant), accounting for 70% of cases, usually originates at the junction of the distal antebrachial fascia and the flexor retinaculum, in the region of the palmar carpal ligament, near the radial aspect of the pisiform at the proximal GC border 15 . It consists mainly of fascia proximally and muscle starting at the level of the GC, extending distally. The average distance from the aADM to the UN at the GC is 0.91 mm. Type II (muscular variant) accounts for 30% of cases, generally arising from the distal antebrachial fascia 13 , and consists entirely of muscle throughout its course. The average distance to the UN is 0.7 mm, and it has greater transverse and anteroposterior dimensions than type I. On MRI, asymptomatic contact or displacement of the ulnar neurovascular bundle is seen in one-third of type II cases. Symptomatic compression is more frequent in this type 13 . Rarely, the aADM passes between the UN and the UA, with the UA superficial and the UN deep with respect to the muscle 13 , potentially predisposing to UA thrombus formation. Morphologically, the muscle is usually unipennate, though bipennate variants exist. High-resolution US and MRI ( Fig. 9 , Fig. 10 ) are useful to characterize the type of aADM and its anatomical relationships, particularly with the UN. Clinical diagnosis of GC compression may remain challenging, even with EMG studies, which can be inconclusive. Surgical resection of the aADM has led to resolution of symptoms in some patients 16 . Nonetheless, despite close proximity between the aADM and the UN, clearly documented symptomatic compression cases remain rare 17 18 19 . Compression of the deep branch of the UA may lead to denervation changes in affected muscles, such as the ADM and aADM 13 . MRI signs of UA compression include vessel enlargement, T2 hyperintensity, or evidence of denervation in the innervated muscles. In certain situations, compression by an aADM may be dynamic and triggered by factors that increase pressure within the GC—such as acute trauma, repetitive microtrauma (e.g., in manual laborers or athletes), or hypertrophy of the aADM 13 19 20 .

### 5.3. Hypothenar adductor muscle

This muscle was first described in 1996 as located deep with respect to the PBM and inserted into the hypothenar fascia. It is oriented transversely. There are two types. In the first type, the muscle extends from the U-shaped aspect of the distal portion of the transverse carpal ligament and inserts into the hypothenar fascia. It covers and compresses the deep branch of the UN. In the second type, the muscle extends from the periosteum to the ulnar aspect of the base of the hamulus of the hamate and inserts into the deepest proximal hypothenar muscle fascia. It covers and compresses the UN near the motor and sensory branch 21 .

### 5.4. Extensor digitorum brevis manus muscle

**Fig. 11:**
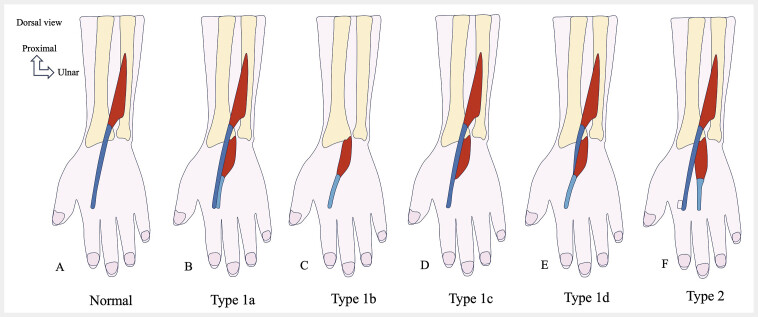
This anatomical variant can mimic a pseudotumor on imaging and must be identified to prevent misdiagnosis. Classification of extensor digitorum brevis manus (EDBM) variants. Schematic drawing showing the extensor indicis (EI, dark blue) and the EDBM (light blue). Normal appearance (drawing **A** ). In type 1 ( **B, C, D, E** ), the tendon inserts into the index finger — the most common variant — subdivided into four subtypes based on its relationship to the EI: type 1a (drawing **B** ): insertion into the index finger via a separate tendon; type 1b (drawing **C** ): insertion into the index finger with an absent EI muscle and tendon; type 1c (drawing **D** ): insertion via a tendon shared with the EI; type 1d (drawing **E** ): insertion into the index finger with a hypoplastic EI. In type 2 (drawing **F** ), the EDBM inserts into the third finger. Figure adapted from: Triantafyllou G, Piagkou M, Paschopoulos I et al. The extensor digitorum brevis manus variability and clinical significance: a systematic review with meta-analysis. Surg Radiol Anat. 2024 Dec 5;47(1):18.

The prevalence of the EDBM is 1.96% 22 . The EDBM most frequently arises from the dorsal wrist capsule in the fourth dorsal compartment of the wrist 23 . It may originate from the posterior radiocarpal ligament, with periosteal attachments to the radius 24 . A classification of EDBM variants has been proposed based on EDBM insertion sites ( Fig. 11 ). In type 1, the tendon inserts into the index finger (prevalence of 1.14%). This is the most common variant. In type 2, it inserts into the third finger (prevalence of 0.19%). The index finger insertion type is divided into four subtypes based on the relationship of the EDBM to the extensor indicis (EI). Type 1a represents insertion via a separate tendon (prevalence of 0.38%), type 1b corresponds to the index finger insertion associated with an absent EI muscle and tendon (prevalence of 0.26%), type 1c corresponds to insertion via a tendon shared with the EI (prevalence of 0.05%), and type 1d corresponds to the index finger insertion coexisting with a hypoplastic EI (prevalence < 0.01%).The EDBM is innervated by a branch of the posterior interosseous nerve 24 . The EDBM is vascularized by a posterior branch of the anterior interosseous artery 24 or the posterior interosseous artery 23 .

**Fig. 12:**
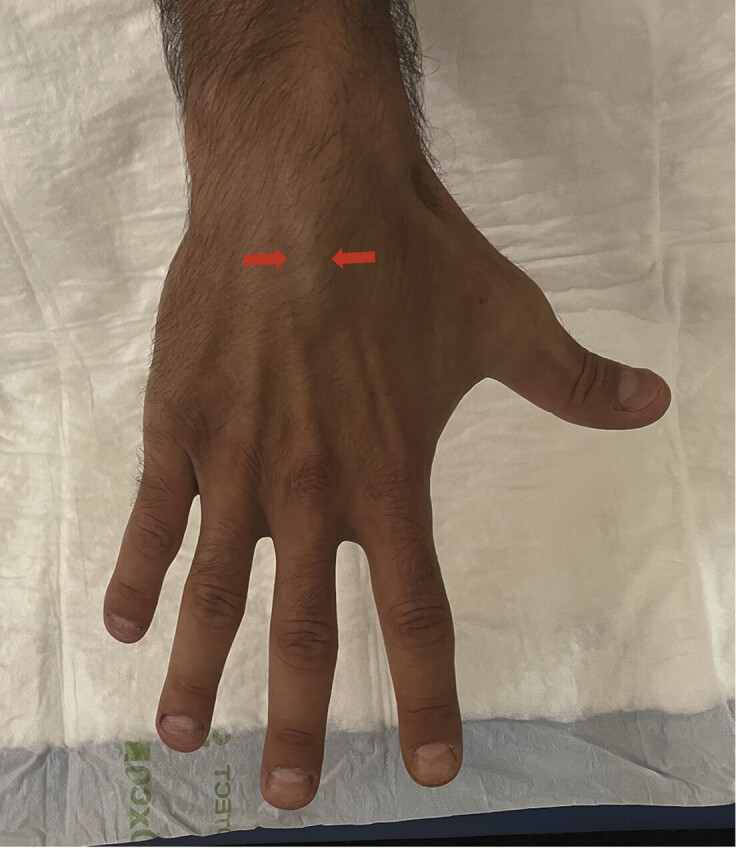
Clinical presentation of a variant simulating a pseudotumor: extensor digitorum brevis manus. Elongated dorsal swelling over the hand between the extensor tendons of the index and middle fingers (arrows).

*Clinical and surgical significance:* this muscle variant classically presents as an elongated swelling on the dorsal surface of the hand between the extensor tendons of the index and middle fingers ( Fig. 12 ). EDBM is mostly misidentified as a dorsal wrist cyst. It can also mimic several situations, i.e., exostosis, tendon sheath cyst, extensor tendon tenosynovitis, hemangioma, or a benign soft-tissue tumor 23 . It may be clinically confused with a humped carpus or an accessory stylodeum bone. The EDBM may be an option to restore thumb extension after post-traumatic extensor pollicis longus injury, particularly when the EI is absent 25 . In some cases, the EDBM may be asymptomatic or cause pain and swelling of the back of the hand. This symptomatology may be more common in the case of manual work and hand dominance 26 . Treatment of a symptomatic EDBM is initially conservative, with immobilization, anti-inflammatory drugs, and occupational therapy 27 . Injection of botulinum toxin into the muscle belly has been suggested 28 . If conservative treatment fails, surgical treatment is performed. If the EDBM is the only finger extensor, careful debridement can be performed to preserve the tendon attachments. When the primary extensor is preserved, excision could be recommended 27 .

**Fig. 13:**
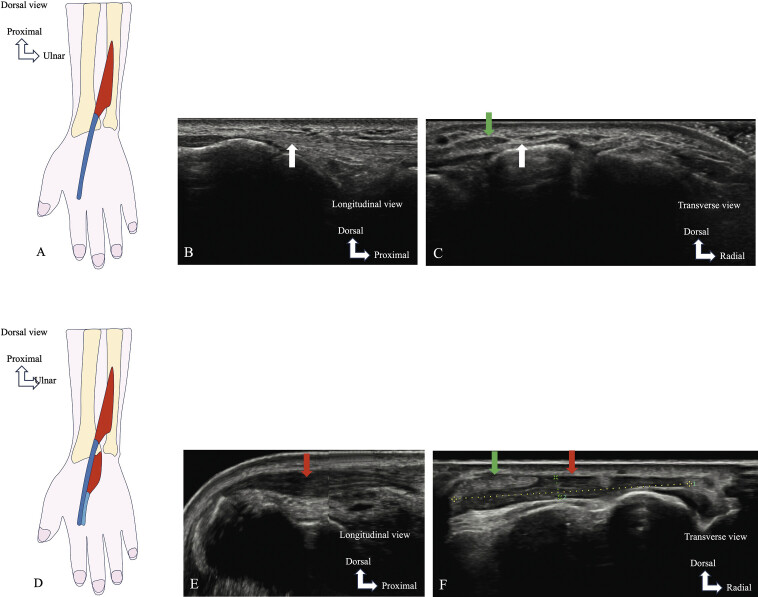
Schematic drawing ( **A** ) showing the normal appearance of the extensor indicis (EI, dark blue). Longitudinal ( **B** ) and axial ( **C** ) ultrasound images show the absence of muscle tissue in the dorsal compartment of the carpal bones (white arrow). Extensor tendons are indicated (green arrow in **C** ). Schematic drawing ( **D** ) and ultrasound images ( **E** : longitudinal, **F** : axial) show the variant simulating a pseudotumor: extensor digitorum brevis manus (light blue), seen as a homogeneous muscle-like dorsal swelling of the wrist (red arrows in **E** and **F** ). Extensor tendons are also visible (green arrow in **F** ).

**Fig. 14:**
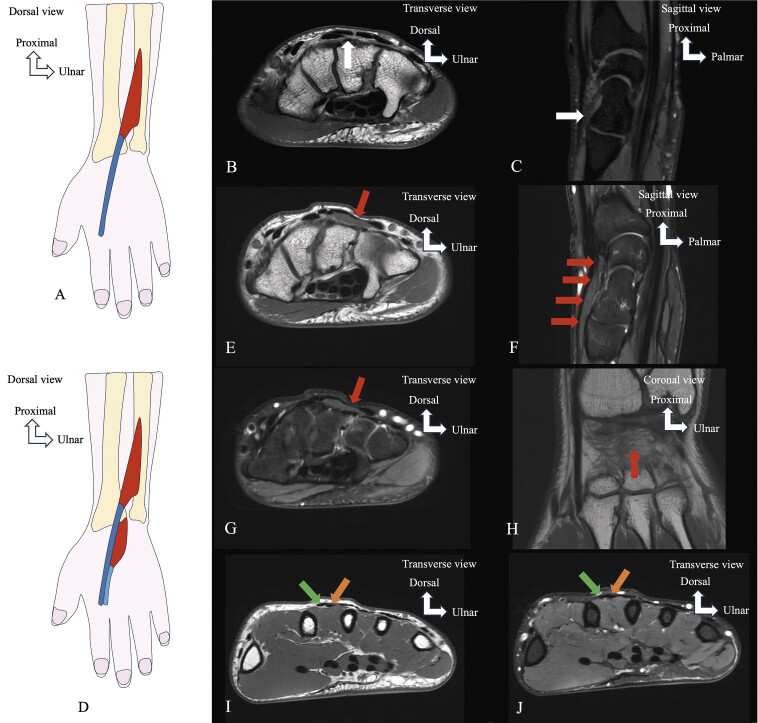
Schematic drawing ( **A** ) showing the normal anatomy of the extensor indicis. MRI images ( **B** : axial T1-weighted TSE, **C** : sagittal PD fat-saturated) show no muscle structure in the dorsal carpal compartment. Schematic drawing ( **D** ) and MRI ( **E–J** ) illustrate a type 1 variant of the extensor digitorum brevis manus (EDBM) simulating a pseudotumor. Axial T1-weighted TSE ( **E, I** ), sagittal PD fat-saturated ( **F** ), axial PD fat-saturated ( **G, J** ), and coronal T1-weighted TSE ( **H** ) show the EDBM (red arrows) originating from the dorsal capsule of the wrist within the fourth dorsal compartment, inserting via a common tendon with the extensor indicis (EI) (green arrows in **I** and **J** ). The extensor digitorum is also identified (orange arrows in **I** and **J** ).

*US* is used to determine the diagnosis ( Fig. 13 ). Dynamic US with active finger extension against resistance could improve the diagnosis of EDBM 29 . One case of a cyst in the EDBM was detected by US 30 . This is an interesting technique because it allows for a contralateral study due to the frequent occurrence of EDBM with bilateral topography. *MRI* ( Fig. 14 ) easily identifies EDBM and allows it to be differentiated in pseudotumoral presentations [31, 23, 32].

### 5.5. Variants of the lumbrical muscles

**Fig. 15:**
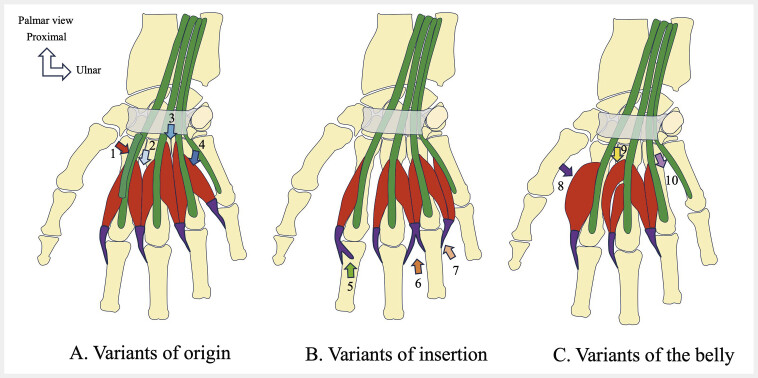
Schematic drawings showing the different variants of the lumbrical muscles. **A** . Variants of origin. Accessory origin from the tendon of the flexor digitorum superficialis muscle (1) (L1), bipennate origin (2) (L2), proximal origin (3) (L3), unipennate origin (4) (L4); **B** . Variants of insertion. Accessory insertion onto the proximal phalanx (5) (L1), split insertion (6) (L3), misplaced insertion (7) (L4); **C** . Variants of the belly. Hypertrophy (8) (L1), accessory belly in the hand (9) (L2), absence (10) (L4). Figure is based on: Belbl M, Kachlik D, Benes M et al. Variations of the lumbrical muscles of the hand: Systematic review and meta-analysis. Ann Anat. 2023 Apr;247:152065.

**Fig. 16:**
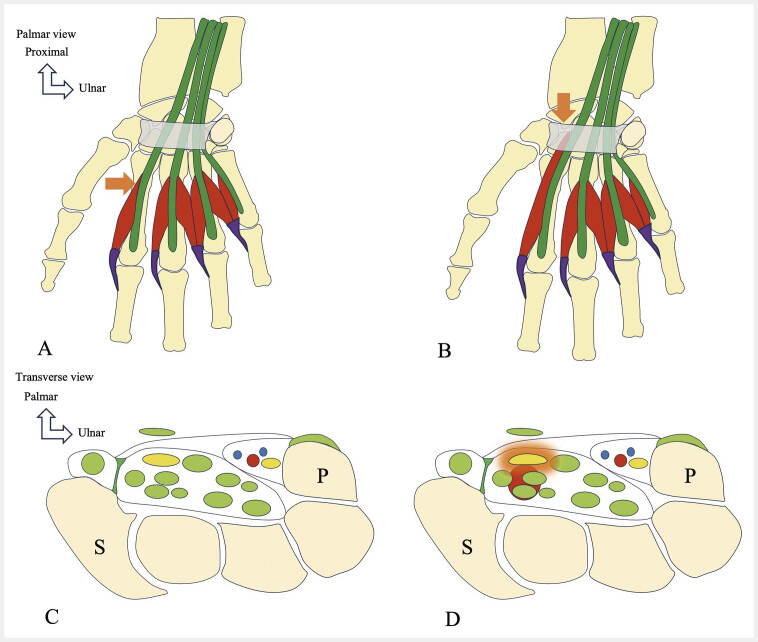
Proximal origin of the first lumbrical muscle within the carpal tunnel leads to median nerve compression and carpal tunnel syndrome. Schematic drawing showing a proximal origin of the first lumbrical muscle (L1) within the carpal tunnel, compressing the median nerve. Normal appearance of the carpal tunnel ( **A, C** ), with the origin of the lumbrical muscles located outside the tunnel. This anatomical variant ( **B, D** ) results in median nerve compression. It may simulate a space-occupying lesion and must be included in the differential diagnosis of idiopathic carpal tunnel syndrome.

**Fig. 17:**
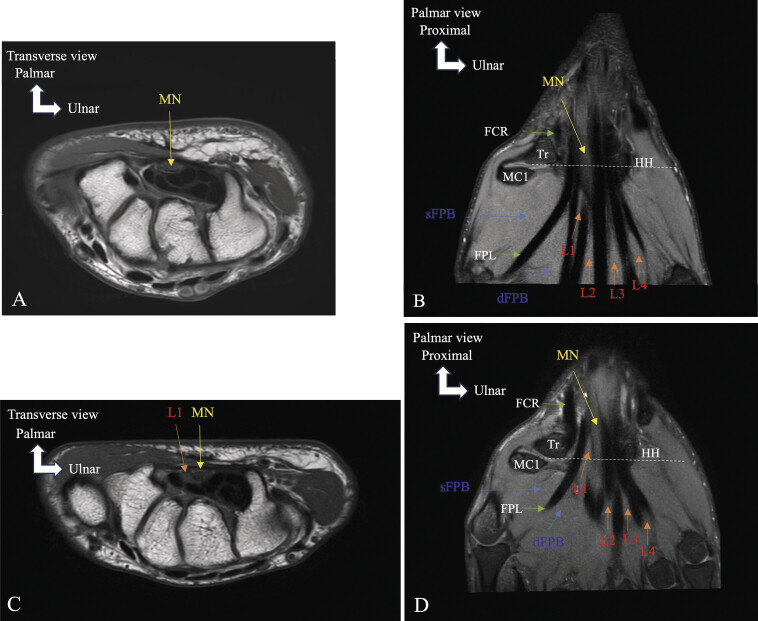
Proximal origin of the first lumbrical muscle within the carpal tunnel induces median nerve compression and carpal tunnel syndrome. Axial MRI in T1 TSE ( **A, C** ), and coronal MRI in PD FS ( **B, D** ). The normal carpal tunnel ( **A, B** ) contains only flexor tendons and the median nerve. Normally, all lumbrical muscles originate distally and outside the carpal tunnel, beyond its distal edge ( **A, B** ). In this variant ( **C, D** ), the first lumbrical muscle arises within the carpal tunnel, increasing pressure within this confined space and compressing the median nerve. Abbreviations : median nerve (MN), flexor carpi radialis (FCR), flexor pollicis longus (FPL), superficial flexor pollicis brevis (sFPB), deep flexor pollicis brevis (dFPB), trapezium (Tr), hamulus of the hamate (HH), first metacarpal (MC1), first lumbrical muscle (L1), second lumbrical muscle (L2), third lumbrical muscle (L3), forth lumbrical muscle (L4).

Multiple variants of the LM exist ( Fig. 15 ). The most common variations for each muscle are an accessory belly for the first lumbrical (L1) (3.8%), a variable origin for the second lumbrical (L2) (7.7%), variable innervation for the third lumbrical (L3) (12%), and a variable insertion for the fourth lumbrical (L4) (5.8%) 33 . L1 variations include a proximal origin variant (2.5%), an accessory muscle belly (3.8%), and muscle hypertrophy (2.1%) 33 . Variations in the insertion and innervation of L1 are very rare (<0.1%). Variations in L2 include variant origin (7.7%), variable proximal or bipennate origins 33 , variant innervation (1.4%), accessory belly (1.2%), and variant insertion (<0.1%). L3 variations involve changes in innervation (12%), exclusive or accessory innervation by the MN, insertion variations (7.9%) such as split or displaced insertions, proximal origin (1.8%), or absence of L3 (0.3%) 33 . L4 variants include split and displaced insertions (5.8%), variations in origin (2.8%), unipennate or proximal origins, absence of L4 (1.1%), and innervation variants (0.1%) 33 . CTS is the most common disease associated with variations of the LM 34 , due to LM incursion, which significantly increases pressure within the CT ( Fig. 16 , Fig. 17 ).

The lumbrical muscles may contribute to increased pressure within the CT due to hypertrophy, accessory muscles, or a proximal origin 35 . Such variations should be systematically evaluated in patients with CTS, as a more proximal origin of these muscles can predispose to their intrusion into the CT during finger flexion and contribute to CTS development 36 37 . In severe CTS, compression of the MN may impair L1 and L2 function, affecting precision pinch tests 35 . Lesions affecting the LMs and IOM, especially in deep transmetacarpal injuries generally not repaired, can lead to impairment of fine hand movements 33 . For these reasons, the use of LMs for muscle flaps requires careful consideration 35 .

### 5.6. Distal variants of the flexor digitorum superficialis muscle and accessory flexor digitorum superficialis muscle of the index finger

**Fig. 18:**
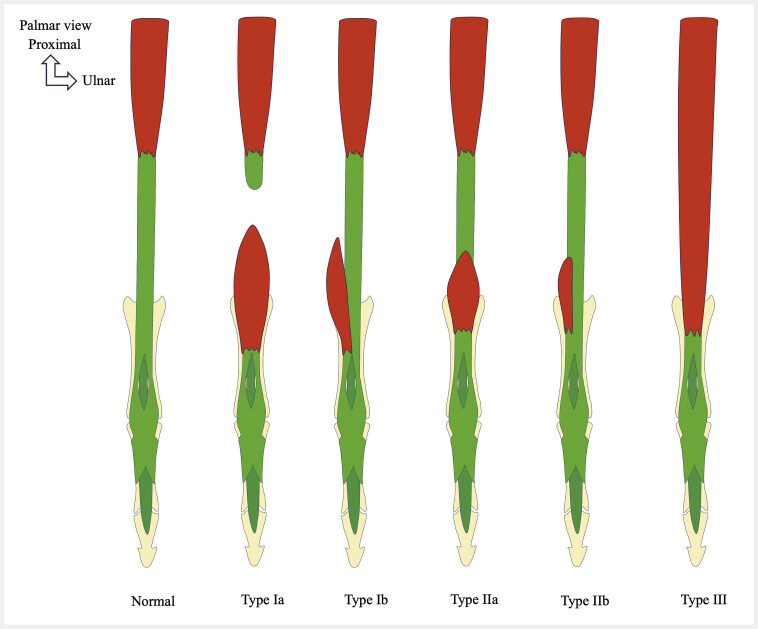
Palmar flexor digitorum superficialis anomalies contribute to carpal tunnel syndrome by increasing pressure within the tunnel. Classification of palmar flexor digitorum superficialis anomalies. Type I, “amphibian” type, characterized by the presence of an intrinsic flexor digitorum superficialis brevis: Type Ia, complete replacement of the normal flexor digitorum superficialis longus tendon by the brevis; type Ib, brevis coexisting alongside the longus tendon. Type II, presence of an additional muscle belly in the hand: Type IIa, interrupted flexor digitorum superficialis tendon with muscle belly; Type IIb, muscle belly alongside the tendon in the hand. Type III, muscle belly located in the palm. Figure adapted from: Bhat W, Davis CR, Akali A et al. Painful, palpable and pathological: anomalous flexor digitorum superficialis brevis in the palm, comparative anatomical context, and an updated classification of anomalies of the flexor digitorum superficialis. J Hand Surg Eur Vol. 2014 Jan;39(1):101–6.

Variations of the flexor digitorum superficialis muscle can be classified as muscle belly abnormalities or tendon arrangement abnormalities 38 . Muscle belly variants may present as a mass or cause symptoms related to MN compression 39 and are categorized into three types 40 : a short muscle arising from the carpal ligament, an elongated muscle belly extending through the CT, or a digastric muscle with an additional muscle belly replacing part of the tendon in the palm. These abnormalities most commonly involve the flexor digitorum superficialis of the index finger in 80% of cases, with a higher prevalence in women 38 . The little finger is affected in 15% of cases, and the middle and ring fingers in 5% 41 . Tendon abnormalities generally produce few clinical symptoms 38 . An updated classification of palmar flexor digitorum superficialis anomalies has been proposed ( Fig. 18 ).

**Fig. 19:**
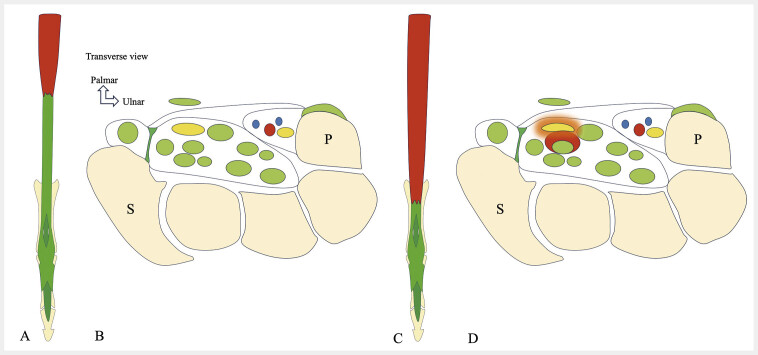
An accessory flexor digitorum superficialis muscle of the index finger increases pressure in the carpal tunnel, leading to carpal tunnel syndrome. Schematic drawing showing an accessory muscle of the index finger. Normally ( **A, B** ), pressure inside the carpal tunnel remains normal. Distal extension of this accessory muscle ( **C, D** ) elevates pressure within the tunnel and triggers carpal tunnel syndrome.

**Fig. 20:**
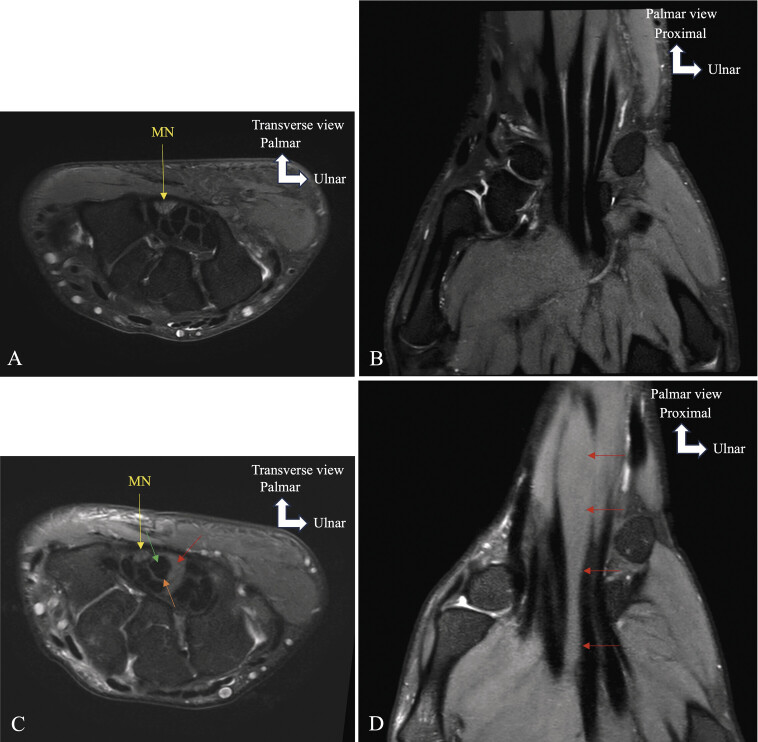
Distal extension of an accessory flexor digitorum superficialis muscle of the index finger into the carpal tunnel increases pressure, resulting in carpal tunnel syndrome. Axial MRI ( **A, C** ) and coronal MRI ( **B, D** ) in PD FS. The normal carpal tunnel ( **A, B** ) contains only flexor tendons and the median nerve. Normally, the distal end of all flexor digitorum superficialis muscles terminates proximal to the carpal tunnel ( **A, B** ). In this variant ( **C, D** ), the muscle belly extends distally beyond the carpal tunnel, increasing pressure within the tunnel and potentially inducing carpal tunnel syndrome. The accessory muscle is indicated by red arrows ( **C, D** ), the flexor digitorum superficialis of the index finger by the orange arrow ( **C** ), and that of the middle finger by the green arrow ( **C** ).

The accessory flexor digitorum superficialis muscle of the index finger causes flexion at the proximal interphalangeal joint 42 and is innervated by the MN via its branch to the L1 42 . This accessory muscle can be mistaken for soft-tissue tumors such as tendon sheath tumors, lipomas, cysts, or vascular malformations 42 . A variant musculature of the flexor digitorum superficialis that increases pressure within the CT is a rarely described cause of MN compression ( Fig. 19 , Fig. 20 ), estimated to affect approximately 1.3% of patients with CTS 34 . Muscle belly variants of the accessory flexor digitorum superficialis muscle causing symptoms are rare 43 44 . Electromyography and/or neuromuscular US may assist in diagnosis or surgical planning but are not mandatory. While electromyography was historically the gold standard for CTS assessment, neuromuscular US has recently demonstrated comparable sensitivity and specificity 45 .

The accessory flexor digitorum superficialis brevis of the little finger is rare, and may be asymptomatic 46 or cause symptomatic compression of the MN 47 .

*The accessory flexor digitorum profundus indicis is also rare* 48 *.*

Muscle variants in the hand exhibit wide anatomical diversity, ranging from frequently observed forms to rarer clinical cases. Some, such as variants of the abductor and flexor muscles of the little finger, the LMs, or the aADM, are well documented and recognized in clinical practice 1 5 13 33 . Their identification is essential due to their potential impact on local biomechanics and possible symptomatic involvement. The EDBM as well as its role in the differential diagnosis of hand masses is extensively documented 22 23 26 31 . Its recognition is crucial to avoid diagnostic errors and to guide appropriate management 29 30 32 . Distal variants of the flexor digitorum superficialis, including accessory muscles of the index finger, are well described and associated with known clinical manifestations, facilitating better diagnostic and therapeutic orientation 38 42 44 . Other variants, such as the hypothenar adductor muscle, are rarer and often reported as isolated cases, warranting further studies to better understand their clinical significance 21 . It is, therefore, important to clearly distinguish between well-established variants and rare cases to assist clinicians in correctly interpreting imaging and guiding treatment decisions. Thorough understanding of classic variants limits unnecessary interventions and improves the comprehension of functional disorders, while ongoing documentation of rare variants enhances knowledge and management of these anomalies.

This review has certain methodological limitations. Although it is based on the experience of a single center, the illustrations and clinical cases presented come directly from our practice, providing a concrete representation of anatomical variants of the IMH. However, this is not an exhaustive case series. Further studies, including additional observations, would be necessary to increase and consolidate knowledge in this field.

The precise differentiation of muscle bellies specific to each muscle can be challenging on imaging, particularly due to anatomical fusions or the sometimes limited spatial resolution of MRI, related to the small size and complex orientation of certain structures. The tendons of the IMH are subject to similar constraints. Moreover, interindividual variability in imaging appearance was not explored in this study.

Designed from a didactic and updated perspective, this review relies on our clinical experience as well as a comprehensive analysis of recent literature. It aims to provide radiologists and clinicians with a clear and accessible reference framework while encouraging the development of broader and more systematic future research. In conclusion, this study offers a structured overview of the IMH, providing a solid foundation for clinical application and future anatomical and radiological investigations.
